# Properties and Applications of Random Lasers as Emerging Light Sources and Optical Sensors: A Review

**DOI:** 10.3390/s23010247

**Published:** 2022-12-26

**Authors:** Dongqin Ni, Moritz Späth, Florian Klämpfl, Martin Hohmann

**Affiliations:** 1 Institute of Photonic Technologies (LPT), Friedrich-Alexander-Universität Erlangen-Nürnberg (FAU), Konrad-Zuse-Straße 3/5, 91052 Erlangen, Germany; 2 Erlangen Graduate School in Advanced Optical Technologies (SAOT), Friedrich-Alexander-Universität Erlangen-Nürnberg (FAU), Paul-Gordan-Straße 6, 91052 Erlangen, Germany

**Keywords:** random lasing, light scattering, light source, optical sensor, stimulated Raman scattering

## Abstract

In a random laser (RL), optical feedback arises from multiple scattering instead of conventional mirrors. RLs generate a laser-like emission, and meanwhile take advantage of a simpler and more flexible laser configuration. The applicability of RLs as light sources and optical sensors has been proved. These applications have been extended to the biological field, with tissues as natural scattering materials. Herein, the current state of the RL properties and applications was reviewed.

## 1. Introduction

The effect of light being able to be scattered multiple times and amplified at the same time to produce stimulated emission and lasing was proposed in 1966 by Ambartsumyan et al. when one mirror of a Fabry–Pérot (FP) cavity was replaced by a scattering surface [1]. Already two years later, Letokhov explored the diffusion equation to theoretically predict a laser-like emission in a randomly scattering gain medium [2]. This laser-like emission was first experimentally evidenced by Markushev et al. in 1986 in a powder system without mirrors [3]. Afterwards, in 1994, Lawandy et al. observed the same phenomenon in a liquid system, where TiO${}_{2}$ powders as scatterers were dispersed in a Rhodamine 640 (Rh640) laser dye solution [4]. In their experiments, the spectra exhibited typical lasing characteristics with an increased emission intensity and narrowed spectral linewidth when the pump energy was increased over a certain energy range, i.e., over the lasing threshold. In 1995, this kind of laser was named a random laser (RL) due to the configurations of random scatterers and the laser-like emissions compared to the conventional lasers [5].

In an RL, multiple light scattering instead of conventional mirrors contributes to the optical feedback. The multiple scattering can currently be obtained from various media, such as polymers [6,7], crystalline materials [8,9], semiconductor materials [10,11], scattering micro-/nano-particles [12,13] or a combination of the above scattering systems [14,15,16]. In addition, RLs have been achieved in biological materials such as abalone shell [17], silk [18], eggshell membranes [19], leaves [20,21], wings of butterfly/moth/cicada [22,23,24], animal tissues (chicken breast [25], cortical bone [26], pig head [27], mouse brain [28], mouse uterine [29]) and human tissues (colon [30], thyroid [31] and breast [32,33,34]). Light can undergo scattering because of the presence of the dielectric constant variations in the above various complex systems. This variation at a microscopic scale gives rise to the macroscopic multiple scattering of the light transport.

**Figure 1:**
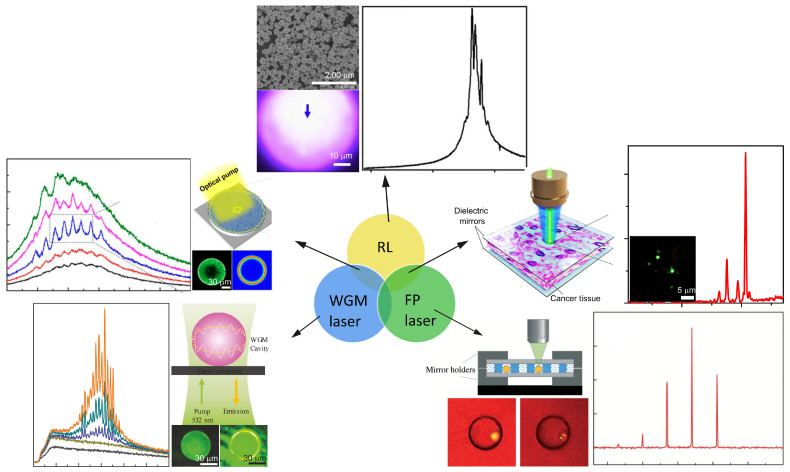
Different optical feedback mechanisms in RL, WGM laser and FP laser. The corresponding setups, lasing cavities and typical lasing spectra are shown beside. RL: top. Adapted from Ref. [35] with permission from IOP Publishing under CC BY 3.0 license. WGM laser: left bottom. Adapted from Ref. [36] with permission from WILEY-VCH under CC BY 4.0 license. FP laser: right bottom. Adapted from Ref. [37] with permission from Royal Society of Chemistry. RL in combination with WGM laser: left top. Adapted from Ref. [38] with permission from MDPI under CC BY 4.0 license. RL in combination with FP laser: right top. Adapted from Ref. [39] with permission from Springer Nature.

Although random scatterers provide a flexible optical feedback, this configuration also makes the RL a highly open and lossy system. This hinders the further applications of RLs regarding the advantages of lasing properties. Therefore, the multiple scattering of RLs combined with other feedback mechanisms is currently being developed. Figure 1 summarizes the difference among distinct optical feedback mechanisms. In a whispering gallery mode (WGM) cavity (see Figure 1 left bottom), light is guided along the interior wall of a spherical cavity. The optical feedback stems from the total internal reflection due to the refractive index difference between the cavity and the surrounding medium. In an FP cavity (see Figure 1 right bottom), the optical feedback arises from the reflection of two reflectors, such as mirrors. Since configurations of WGM and FP cavities are well-defined, lasing modes are regular, as shown in Figure 1 left and right bottom. In a pure RL, the lasing modes are more like random spikes (see Figure 1 top). This review includes RL systems with a feedback mechanism of pure multiple scattering, as well as hybrid feedback mechanisms. For instance, this also includes RLs in combination with the WGM cavity (Figure 1 left top) and RLs in combination with the FP cavity (Figure 1 right top).

One might confuse the concept of an RL with other categories of light amplification, such as superfluorescence (SF), superluminescence (SL)/amplified spontaneous emission (ASE) and superradiance (SR). All of them are mirror-less light amplifications and exhibit threshold-like behaviors [40]. A more detailed explanation and comparison of SF, SL/ASE and SR refers to [40]. The RL is distinguished from the rest as the RL is mirror-less but not feedback-less [41,42]. The random medium induces the essential feedback for light amplification through the multiple scattering, whereas the features and disorders of the random medium can be represented by the RL emission properties [42].

Compared to conventional lasers, RLs are simple, flexible, relatively small and relatively economical. The emissions of RLs have a relatively low coherence. The RL wavelength can be tuned as well. These advantages benefit RLs in specific applications such as light sources. For instance, stretchable/bendable RLs [16,43,44,45,46] and wavelength-tunable RLs [43,46,47,48] have been achieved. In addition, RLs have been proved as ideal light sources in speckle-free imaging due to the property of a low spatial coherence [49]. In recent years, lasers are of critical importance in medical and biological fields [50]. In this context, the RLs generated from the biological materials have the potential to compensate for the lack of bio-compatible lasers [20,21,24,51]. Biocompatible lasers are lasers made of biomaterials for biosensing and biomedical applications.

RLs are generated from disordered systems and RLs can be employed as optical sensors to detect variations in the disordered systems. Variations in scatterer concentrations [52,53,54], gain media [53] and refractive indices of solvent [55,56] have been detected through the RL emission. Sensing applications have been extended to the biological field, such as the optomechanical and bio-chemical sensing (nanoscale mechanical sensing in hard [26] and soft [57] tissues, dopamine sensing [58], PH sensing [59] and human antibody IgG sensing [60]), tissue differentiation [27], cell differentiation [28,61], cancer diagnosis [30,31,32] and cancer therapy [29,33]. A sensing scale of RLs down to the cell level has recently attracted an increasing interest [34,51,62].

In the last two decades, several review articles have provided an extensive insight of the physics [41,42,63,64,65,66,67,68] and potential applications of RLs [68,69,70,71,72,73]. Along with a deeper physical understanding and rapid development of new materials and new findings [74,75,76], an updated review article seems in high demand. On the one hand, although the experimental observations and applications of RLs have emerged rapidly recently, there seems to be a lack of a systematic description of them. Especially for a reliable sensing application, the intrinsic relationship between the input (e.g., PH variations) and output (e.g., the RL emission properties) parameters should be clearly understood. On the other hand, a summary of the emerging new physics, such as the stimulated Raman scattering (SRS) in RLs, which is promising for potential RL applications, is still lacking. Therefore, the aim of this review is mainly to bring a deep discussion of the RL properties and their control approaches, as well as their relative applications.

This review is structured as follows: RL emission properties followed by RL applications are first introduced. It is necessary to understand the RL emission properties before they are applied to practices. The RL applications focus on light sources and optical sensors. This review ends with an outlook for further research topics of RLs.

## 2. RL Emission Properties

RL emission shows certain properties, including the properties of a lasing threshold, peak wavelength and coherence. In addition to the common laser spectral properties, such as the linewidth narrowing and intensity enhancement over the lasing threshold, RL also exhibits properties such as a tunable peak wavelength and tunable coherence. The investigated influence factors of RL emissions vary from author to author [77]. Table 1 summarizes the five general influence factors of the RL emission properties, with examples for each. The details of Table 1 are explained in the following subsections.

### 2.1. Lasing Threshold Characterization and Reduction

The lasing threshold, an important RL property, has been thoroughly investigated. The threshold can be characterized by an increased emission intensity or narrowed spectral line width. In addition to the traditional method in frequency spectra, the RL threshold can also be determined by measuring temporal profiles [95]. The building time of an RL pulse is apparently shortened around the threshold due to the increased gain of the stimulated emission upon crossing the threshold. Another particular RL property is that the shot-by-shot emission intensity fluctuates under the same experimental conditions. This property can be used to assess the lasing threshold too.

A statistical measurement of the fluctuated intensity indicates an intriguing distribution change: a change from Gaussian to Lévy shape at the onset of the RL threshold, thus providing another identifier of the threshold [96]. Interestingly, an intrinsic threshold regime rather than a threshold point was revealed [97]. In this threshold regime, a progressive growth in the coherent field caused by the stimulated emission was observed [97]. Such a progressively smoother transition was more frequently observed in a micro-/nano-laser than in a macroscopic laser [98]. A further statistical analysis of the fluctuation correlation among the above RL spectrum replicas resulted in a replica symmetry breaking (RSB) observation around the threshold [99]. RSB is a state-of-the-art method for predicting a phase transition of a complex system and reveals the interplay between the disorders of and fluctuations in the system [100]. In the case of RLs, the phase transition refers to a transition from a non-lasing to lasing state. The revealed interplay is the one between the multiple scattering and the quantum noises (e.g., spontaneous emission) responsible for the start of the lasing.

In an RL, the peculiar optical feedback and extreme light leakage at boundaries contribute to the inefficient gain–loss balance around the threshold, leading to a high-threshold RL emission. Currently, the research focus of the RL threshold is on its reduction. This is significant because the high-threshold laser emission usually hinders the further applicability of RLs; for example, applications in the biological field where the excitation intensity is required to be low to avoid photo-toxicity to tissues. In this regard, changing the shape of scatterers to increase the surface area [12] or optimizing the size of scatterers [92] can facilitate the scattering efficiency and, therefore, lower the RL threshold. In other reports, additional disorders were introduced into a spatial distribution of pump light to lower the RL threshold [84,85]. The RL threshold can also be lowered by employing metal nanoparticles (NPs), in which, the surface plasmon resonance can enable a high gain for lasing even at a low excitation intensity [16,60,92,101,102,103]. Furthermore, external optical cavities such as the fiber structure [47,94,104], FP cavity [39,105,106,107,108,109] and WGM cavity [38,110] were proposed to enhance the optical feedback of RLs and eventually reduce the lasing threshold.

### 2.2. Peak Wavelength Shift (Tunability) 

A shifted peak wavelength is usually observed in the spectra of dye-based RLs [111]. In a pure dye solution, the peak wavelength redshift was ascribed to dye aggregates [112] or dye reabsorption [113]. Dye aggregates are formed at higher concentrations, leading to a second spectral band at the longer wavelength side, i.e., redshift [112]. Dye reabsorption happens because of the overlap between the absorption and emission spectra of dyes, known as a secondary inner filter effect [113]. It was reported that when the fluorescent dye without spectral overlap was applied, no redshift due to reabsorption and re-emission was observed in the pure dye solution [113]. The above two effects were employed to explain the most redshift observed in the dye-based RLs [52,114,115]. Depending on the experimental configurations, both enhanced redshift (*relative* redshift) and weakened redshift (*relative* blueshift) are observed. The details are discussed as follows.

#### 2.2.1. Relative Redshift Realization

Increasing scatterer concentrations is a common approach for inducing a relative redshift [52,111]. A stronger scattering strength leads to a longer light dwell path where the fluorescence reabsorption and re-emission effect occurs [115]. Hence, a stronger redshift (*relative* redshift) is expected. In turn, the peak redshift can be used to characterize the light path length [52]. In a further step, the peak shift has the potential for the detection of variations in the scattering or absorption strength. For instance, Ignesti et al. [111] reported a maximum redshift of around 50 nm when increasing concentrations of intralipid in a dye solution. Other approaches, such as using larger microspheres diameters [116] and increasing the laser cavity lengths [117], also facilitate the peak wavelength redshift.

#### 2.2.2. Relative Blueshift Realization

On the contrary, El-Dardirya and Lagendijk [118] demonstrated a blueshift by increasing concentrations of non-fluorescent absorbers in an RL medium. This is expected as the light path in the gain medium is reduced by the absorption. This results in a weak reabsorption and reemission effect (*relative* blueshift). Another blueshift phenomenon was observed in a simulation case when pump energy was well above the lasing threshold. This was explained by the depletion of the ground state, which pulls the peak emission towards smaller wavelengths for a stronger amplification. Intriguingly, increasing scattering can induce a relative blueshift rather than redshift. For instance, the blueshift was observed when increasing concentrations of TiO${}_{2}$ scatterers in Rhodamine B (RhB) [119] or in Rhodamine 6G (R6G) [93] methanol solution. Likewise, Hohmann et al. [52] reported a slight blueshift at a relatively high concentration of intralipid scatterers in an R6G water solution.

The reasons for blueshift when increasing the scattering strength are not clear in the literature. Hohmann et al. [52] ascribed the blueshift to the falling numbers of optimal lasing microcavities. Because the scattering mean free path $l_{s}$ at relatively strong scattering media is too short to construct optimal microcavities, the total light path length is reduced so that the blueshift happens. Others [119] believed that the lasing peak was pulled towards the shorter wavelength side to obtain a maximum gain. Bavali et al. [113] provided another aspect to explain the blueshift. As concentrations of scatterers increase, the overlapping area of dye absorption and emission spectra shrinks such that the redshift is saturated. A further increment of scatterers in saturated media gives rise to the encirclement of dye molecules. This has an equivalent effect to reducing dye concentrations, so blueshift occurs due to the weak reabsorption effect. This argument of blueshift might be evidenced in experiments [93,119] where, in a saturated medium with sufficiently high concentrations of dyes, only blueshift occurs when increasing the concentrations of scatterers.

In a summary of Section 2.2, the redshift can be enhanced or weakened depending on the experimental and material properties. If no saturated effects of lasing and dye media play a role, it is the effects of scattering and absorption that determine the light dwell path length. This effect results in fluorescence reabsorption and re-emission, as well as the peak wavelength redshift in RLs. This wavelength shift promises to construct a wavelength-tunable laser source and also promises an optical sensor; for instance, for the scattering strength sensing. The details are introduced in the section of the RL applications.

### 2.3. Coherence

RLs show spatial and temporal coherence similar to a conventional laser. Spatial (temporal) coherence describes the correlation of waves at different points in space (time). On the one hand, an RL temporal coherence measurement was performed in the Michelson (Twyman–Green) interferometer [120,121]. Under the common RL configuration with single-shot excitation, titled mirrors were proposed in the Michelson interferometer to give enough of a path delay along the mirrors for the temporal measurements [120]. On the other hand, the Young’s double slit interferometric scheme was utilized for the measurement of the RL spatial coherence [83,121]. A low spatial coherence of RL emission was reported during the measurement [122]. Regarding the RL coherence, the characteristics of spectral modes are discussed in detail in the following, as well as the corresponding origins of the spatial modes.

#### 2.3.1. Characteristics of Spectral Modes

From the point of view of spectral modes, the observed RL emission is classified into two types. One is an incoherent feedback RL emission characterized by a single continuous spectrum with a linewidth of a few nanometers. The other is a coherent feedback RL emission characterized by a spectrum comprising multiple discrete modes, each with a linewidth of sub-nanometers [63].

The different characteristics of RL spectra have been previously explained by Cao et al. [63] from the macroscopic perspective of the feedback mechanism: the former spectrum is attributed to the incoherent (non-resonant, intensity or energy) feedback where the light propagates along open trajectories. The latter one arises from the coherent (resonant, field or amplitude) feedback where the emitted light returns to the position that it has visited before. An optically closed loop is formed through the coherent feedback mechanism, resulting in light interference and light confinement. Due to the wavelength-sensitive interference effect, only light of certain wavelengths can be confined in such a cavity [63]. This is analogous to the conventional lasers, in which, the cavity changes the frequency of the emission light, as well as the directionality.

The lack of multiple modes in the incoherent feedback RL spectra was assumed by Cao et al. [63] to be a lack of lasing cavities or interference. In contrast, another assumption was that the interference in an incoherent feedback RL exists, but the interference effect is averaged out because the lasing cavities overlap in the spatial region [2,41]. It was found that the presence and even the number of multiple modes highly depend on the experimental conditions. For instance, a transition from an incoherent feedback RL to the coherent feedback RL was induced by reducing the pump spot size [80,82] or using a pump pulse duration of a picosecond instead of nanosecond [79,80]. In this way, the mode overlapping in either the spatial domain or time domain is reduced so that the individual modes can be visualized. From the perspective of scattering, increasing the refractive index difference between the scatterers and solvent [123] or increasing the scatterer concentration [63] also gives rise to the easy realization of a coherent feedback RL. The detection angle can also affect the spectral mode visualization. The disappearance of spectral modes was observed when the detection angle was increased on a polymeric capillary system doped with quantum dots [124].

Other effects, such as mode repulsion and coupling, can also change the RL spectral modes profile [81,125,126]. As a complex system, RLs are also suitable for the generation of non-linear optics, which, in turn, induces alterations to the RL spectral modes [127]. One of the non-linear optics, the stimulated Raman scattering, is introduced and discussed later.

#### 2.3.2. Characteristics of Spatial Modes

To understand the origin of the spectral modes, the spatial modes of the system need to be studied initially. The spatial modes can be localized modes when the scattering is strong enough in the Anderson localization regime [88,128]. (Light localization originates from the wave interference between multiple scattering paths. To distinguish from the weak localization in the light diffusion regime, the wave interference influence in the Anderson localization regime—in which, the light diffusion is absent due to the extremely strong scattering—is also called strong localization [129].) However, most of the RL systems are in the diffusive regime. In a passive (without gain) diffusive sample, the extended modes cover the entire system and overlap with each other [41,66]. Due to the overlapping, it is more likely that extended modes are averaged out, leading to a continuous RL spectrum. This is the characteristic of incoherent feedback RLs. A numerical model using light diffusion with gain can explain and predict this phenomenon [2,5]. However, this model does not predict multiple modes since the phase of light field and interference effect are neglected here. Therefore, other models have been introduced to describe how the spatial modes influence the spectral modes in the diffusive regime.

**Figure 2:**
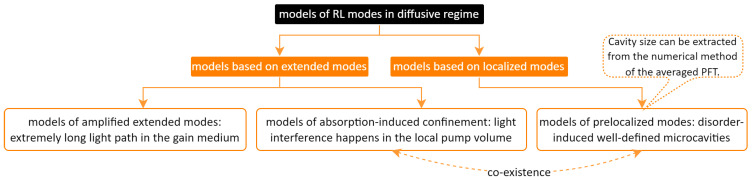
Summary of the models of RL spatial modes in the light diffusive regime.

Figure 2 summarizes the RL modes models in a light diffusive regime. These models can mainly be divided into two groups: one is based on extended modes, such as the model of amplified extended modes [130] and the model of absorption-induced confinement [131]; the other one is based on anomalously localized modes, such as the model of prelocalized modes [132,133]. The detailed explanations are given as follows.

##### Amplified Extended Modes

In the simulation from Mujumdar et al. [130] using the Monte Carlo technique, lasing modes are amplified extended modes. They argued that certain single spontaneous emissions experience extremely long light paths by chance and are consequently amplified to form multiple modes. These extremely long light paths are rare in a passive system and therefore distinct in the RL spectra once they are selected by gain. The author also used the model to explain another experimental phenomenon, where the positions of multiple modes are different in the single-shot excitation. In other words, the multiple modes are random spikes rather than regular discrete peaks. They ascribed the chaotic behavior to the inherent randomness of spontaneous emission.

##### Absorption-Induced Confined Modes

In the numerical study of Yamilov et al. [131] using the finite-difference time-domain method, light confinement is attributed to the optical absorption, e.g., reabsorption from Rhodamine dyes, which limits the number of extended modes to lase. More specifically, the reabsorption effect can suppress the feedback from the unpumped part such that the lasing modes can be confined in the pumped volume. Since the spatial averaging effect is diminished by local pumping, the modes are present on the spectrum. The lasing modes within the effective volume are still the same extended modes as those in a passive system. However, the appearance of the lasing modes depends on the local pumping, e.g., the pumping spot size. In a rather weak scattering system, a regular FP-like cavity is even formed between the base and tip of the pumping cone [134].

Although the amplified extended modes and absorption-induced modes both originate from the extended modes of passive systems, the feedback mechanisms are different. *In the latter one, a regular lasing cavity is formed due to the absorption outside the pump area leading to the correlated spectral modes, whereas no optical cavities exist in the former case and random spikes are formed due to the intrinsic randomness of spontaneous emission.* Thereby, some authors [134,135,136] argued that the emission with stochastic spikes is not laser emission but is amplified spontaneous emission [136].

##### Prelocalized Modes and Size Calculation of the Prelocalized Cavity

The scenario of the optical cavity by simply being scattered from one scatterer to another was doubted for the reason that scatterers could also scatter the light out of the cavity and break the closed loop [132]. Hence, other models suggest that multiple modes are still generated from light interference in analogy to the localized modes in the Anderson localization regime, although there are much fewer localized modes than extended modes in the diffusive regime. This anomalously localized mode in the diffusive regime is named the prelocalized mode by Apalkov et al. [132].

Apalkov et al. modeled the scatterers into a ring-shaped waveguide structure, along which, a higher dielectric constant is present and the prelocalized modes are generated [132]. The likelihood of such ring-shaped microcavities with a fixed size crucially depends on the scatterer’s size [132,133]. Specifically, when the scatterer’s size is larger, the probability of cavity formation is enhanced and the number of scatterers required to form such a cavity is reduced [133]. The ring microcavity here is a simple model for the lasing cavity that is formed due to disorders, which is the same as the simplified model of the FP cavity for the absorption-induced cone volume.

The scenario of disorder-induced ring microcavities was proved by Polson et al. in π-conjugated polymer films with [6] or without [137] TiO${}_{2}$ doping. In these films, the inhomogeneity of the film thickness contributes to the long-range fluctuation in the refractive index. The refractive index difference between the microcavity and surrounding space further facilitates the scattering strength and therefore confines the light. Meanwhile, the existence of short-range disorder induced by individual scatterers suppresses the ability of the most random cavities to trap light, e.g., scatters the light out of the microcavities such that the survived long-range cavities are sparse and consequently almost identical [6]. A highly correlated RL spectrum with regular modes is generated. Although the single-shot RL spectrum is highly reproducible both in powders (Anderson localization regime) [63] and in polymer films (diffusive regime) [6], the lasing mechanism is different. *One emission line from one localized cavity is detected in powder systems, whereas one prelocalized cavity in polymer films generates numerous correlated emission lines due to its larger size.*

Cao et al. [138] investigated the prelocalized modes using spectrally resolved speckle analysis in both a dyed micro-particles polymer system and a semiconductor NPs system. They claimed that the size of the prelocalized mode varies and decreases when the scattering strength moves towards the Anderson localization regime. In other words, the size of the prelocalized mode in the diffusive regime is larger than the size of the localized mode in the Anderson localization regime. This larger cavity size in the diffusive regime agrees with the argument from Polson et al. [6], as mentioned above. In addition, the cavity size is independent of the pump intensity and pump area, indicating that the lasing mode is the intrinsic property of the passive system and only relates to the scattering strength. Note that Cao [64] later pointed out that the model of absorption-induced confinement does not contradict the model of prelocalized modes: *the local pumping does not eliminate the possibility of prelocalized modes and these two models can be simultaneously applied when the prelocalized modes happen within the effective volume induced by the local pumping.*

**Figure 3:**
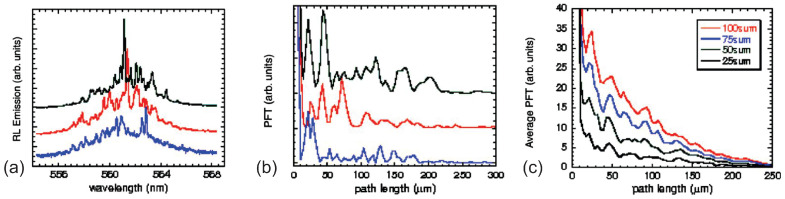
Averaged PFT of RLs in methanol colloid of 1 mM R6G and $8.6\;\times \;10^{9}$ cm${}^{-3}$ TiO${}_{2}$ ($l_{t}\approx 12$ µm). (**a**) Three RL spectra generated by single individual excitation pulses. (**b**) The PFT of RL spectra shown in (**a**). (**c**) Averaged PFT of RL spectra over multiple excitation. The distinct Fourier harmonics are resolved. Adapted from Ref. [25] with permission from American Physical Society.

Since the microcavities have quenched disordered structures, Polson et al. [6] proposed a numerical way, i.e., the power Fourier transform (PFT), to derive the size of the microcavities. In the diffusive regime, the cavities or prelocalized modes are usually hidden by the extended modes, leading to the uncorrelated RL spectral modes; see Figure 3a. Likewise, the corresponding PFT spectra shown in Figure 3b have no correlations with each other [25]. Intriguingly, upon further averaging various PFT spectra, either over the sample area or over the illumination time, some Fourier harmonics are surprisingly not eliminated by averaging. The hidden well-defined cavities are revealed by the positions of these Fourier harmonics as shown in Figure 3c [25]. In detail, the cavity length *L* (or the cavity diameter *D* for the circular microcavity) is calculated via the relation L=dπ/n (or D=2d/n for the circular microcavity). *d* is the first peak position in the averaged PFT and *n* is the refractive index of the random medium [6].

The averaged PFT reveals the universality of the hidden cavities among different scattering systems, such as the R6G infiltrated opals, polymers, scatters and chicken breast [25]. In detail, the size of the dominant cavity can be scaled with the transport mean free path $l_{t}$ among different scattering systems. This indicates again that the microcavity or prelocalized mode is not an artifact; rather, it is induced and only influenced by the multiple scattering despite the varieties of scattering systems [25].

In a summary of Section 2.3, the spectral modes have their origins from the spatial modes or cavities. If the spectral modes are not distorted by the nonlinear effects such as the nonlinear mode competition and the Raman peaks from the stimulated Raman scattering effect, the powerful approach of the averaged PFT can be applied to extract the intrinsic spatial structure of the disordered complex system. For instance, the structure could be the pump spot size or some well-defined physical structures, such as biological cell sizes. The details are introduced later in the section of the RL applications.

To conclude the entire Section 2, the RL spectral properties of the threshold, peak wavelength shift and coherence are described. This is already summarized in Table 1. The physics behind the RL threshold is the gain–loss balance, where the gain is obtained from the stimulated emission, which is enhanced by the multiple scattering. The losses are the spontaneous emission noises and additional absorption losses. The RL peak wavelength shift normally happens in the dye-based random media due to the dye fluorescence reabsorption and re-emission effect. The occurrence and appearance of the RL spectral modes are influenced by the modes overlapping, coupling and competing, both in the spatial domain and in spectra. In this context, on the one hand, varying experimental conditions to control the above three physical mechanisms is possible for the control of the RL emissions; for example, for constructing a modern flexible laser illumination source. On the other hand, analyzing the observed RL emissions to assess the random complex media is also feasible; for example, for optical sensing.

## 3. Emerging RL Physics: Stimulated Raman Scattering 

Recently, narrow peaks in RLs with fixed wavelengths have drawn more and more attention [85,139,140,141,142,143]. These peaks can neither be explained by geometric microcavities, as no such cavities exist in the liquid samples, nor can it be explained by localized modes, as the scattering strength is in the diffusive regime. Furthermore, they cannot be explained by models of RL modes in the diffusive regime as well. The models of absorption-induced confinement and amplified extended modes are not feasible here, since these peaks have fixed wavelengths despite the divergent pump spot sizes. In addition, the wavelengths of peaks are fixed in various systems with distinct scattering strengths. This contracts the universality observed in the model of prelocalized modes: the cavity size should be varied and scaled with the scattering length. In this circumstance, there might be another light–matter interaction besides the multiple elastic scattering.

**Figure 4:**
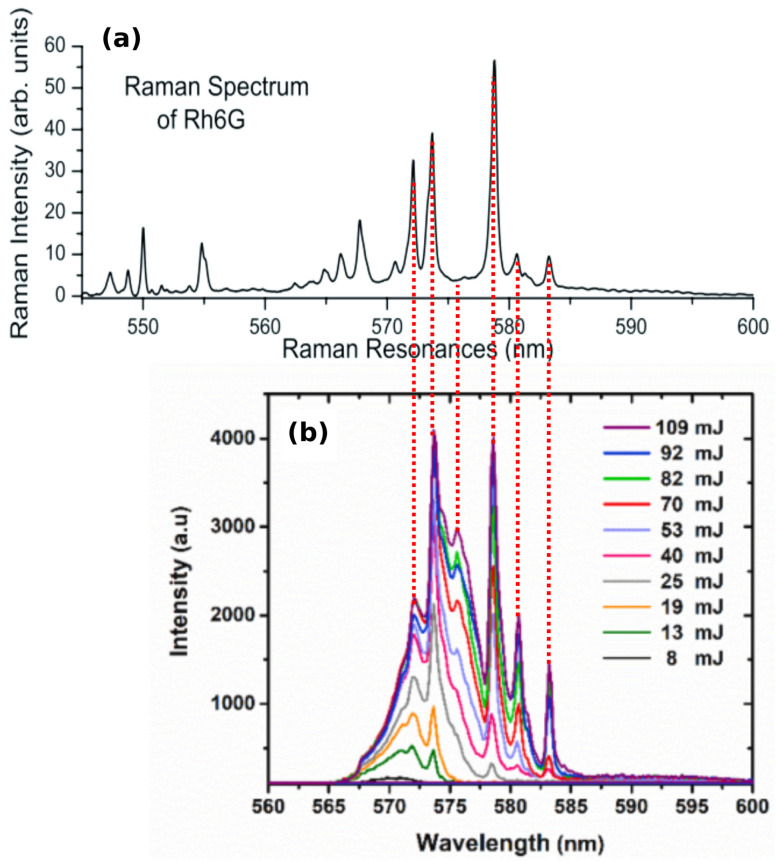
(**a**) Spontaneous Raman spectrum of R6G with excitation wavelength of 532 nm. Adapted from Ref. [141] with permission from IOP Publishing. (**b**) Stimulated Raman scattering signals generated from an RL medium consisting of ZnO nanospheres and R6G methanol colloid in flask, pumped by a laser with wavelength of 532 nm, pulse duration of 7 ns and pump spot of 3 mm. Adapted from Ref. [140] with permission from Elsevier.

The stimulated Raman scattering (SRS) was paid attention. The fixed peaks highly correspond to the Raman lines of fluorescent dye R6G, which is used as the gain medium in RLs, the spectrum of which is shown in Figure 4b. Specifically, compared to the spontaneous Raman spectrum of R6G in Figure 4a, Raman lines at around 572.3, 574, 575.8, 579, 581 and 583.4 nm are probed in the RL spectra. In principle, SRS occurs when the frequency difference between the pump laser and Stokes laser matches the characteristic molecular vibration [144]. Here, the Stokes laser corresponds to the RL. Considering that a broadband RL spectrum coincides with multiple Raman lines, a broad Raman spectrum with rich chemical information instead of a single Raman line could be obtained. In comparison to the standard broadband SRS with two pump lasers [144], this RL-probed broadband SRS has advantages of simple configurations, as the cascade excitation of the RL and SRS is realized by one single pump laser. When the pump intensity is below the lasing threshold of the RL (no RL), the Raman lines from the spontaneous Raman scattering are possibly too low to be pronounced from the fluorescence background. It is worth noting that SRS also occurs in a random Raman laser (RRL) [145]. The difference is that Raman gain is used as the primary gain in the RRL, whereas the fluorescence provides the primary gain to generate the RL and further triggers the SRS in RL-SRS coupled emission.

Raman lines in RL spectra did not attract much attention in the literature in the last two decades. One reason behind this is that the RL-SRS coupled lasing spectrum was misunderstood as a coherent feedback RL. In addition, Raman lines were usually hidden in the spikes in certain conditions, such as picosecond pumping. Therefore, a literature survey of the possible Raman lines in published RL spectra is necessary and was carried out prior. This is summarized in Table A1 in the Appendix A, with the focus on the R6G dye, a common gain medium for RLs.

The observation of Raman lines opens a new perspective of RLs and might extend RL applications in noninvasive chemical probing in the turbid medium; for instance, for the assessment of bone quality during oral surgery [146] and for remote explosives identification for security screening applications [147]. Compared to the conventional Raman spectroscopy, the RL-SRS spectroscopy advances the shorter measurement time and higher signal-to-background ratio due to SRS and, meanwhile, advances the deeper penetration depth due to the multiple scattering in RLs.

## 4. Applications of RLs

RLs have been employed as light sources that exhibit excellent bio-compatibility and flexibility in comparison to the conventional laser light. The most extensive application of RLs as a light source is for speckle-free imaging due to their low spatial coherence. In the meantime, RLs were widely applied in the biological field, especially for optical sensing.

### 4.1. RL as a Light Source 

#### 4.1.1. Bio-Compatible RLs

**Figure 5:**
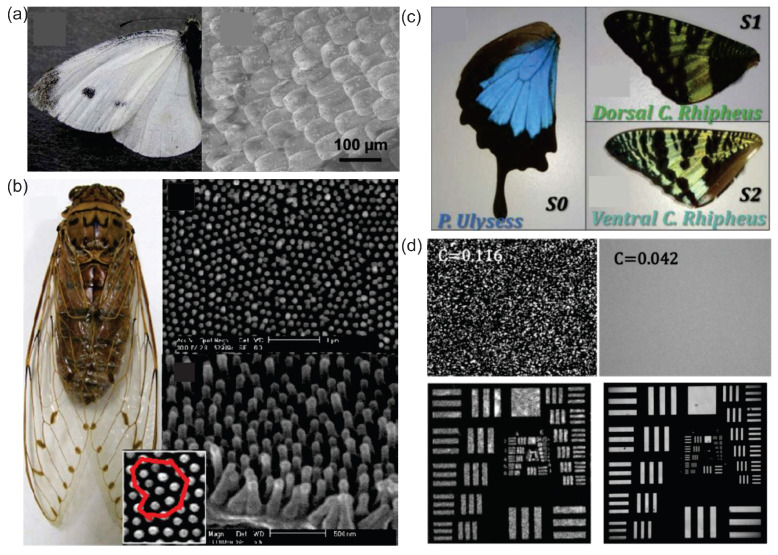
Bio-compatible RLs. (**a**) Picture of a *Pieris canidia* butterfly and its low magnification SEM image of the wing. Adapted from Ref. [22] with permission from Springer Nature under CC BY-NC-ND 4.0 license. (**b**) Picture of the *pomponia imperatoria* cicada and its different magnification SEM images of the wing. Adapted from Ref. [23] with permission from Elsevier B.V. (**c**) Pictures of a lower wing of *P. Ulysses* (S0), an upper dorsal wing of *C. rhipheus* (S1) and an upper ventral wing of *C. rhipheus* (S2). (**d**) Scattering images of a Q-switched Nd:YAG laser with 532 nm wavelength (upper left) and RL of S1 (upper right) without imaging object. The lower row shows the images of 1951 US AF resolution test chart illustrated by a Q-switched Nd:YAG laser with 532 nm wavelength (lower left) and by the RL of S1 (lower right). Adapted from Ref. [24] with permission from Optical Society of America.

RLs can be used to sense vibrations in biological tissues. In turn, biological tissues can be employed to manufacture RLs. The realization of RLs in biological tissues is attributed to the multiple scattering caused by the disorder distributions of bio-nanostructures. For the first time, Siddique et al. [148] demonstrated an RL emission in optically pumped chicken tissues and pig fat samples both treated with Rh640 dye solution. Since then, tissue-based RLs are reported in diverse biological tissues such as Pieris canidia butterfly wing [22] and pomponia imperatorial cicada wing [23]; see Figure 5a,b. The former one was embedded with ZnO NPs and the latter one was coated with an organic-dye-doped polymer as gain media. Such an RL compensates for the lack of biocompatibility in the laser field and paves the way for designing bio-controllable photonic devices.

Moreover, this bio-compatible RL proved to be an ideal light source for speckle-free imaging [21,24]. From the wings of a moth (Figure 5c), the spatial coherence of the RL (Figure 5d upper right) is lower than that from a conventional Q-switched Nd:YAG laser (Figure 5d upper left). As a consequence, the image of the 1951 US Air Force (AF) resolution test chart illustrated by the RL (Figure 5d lower right) shows fewer speckles than that illustrated by a conventional laser (Figure 5d lower left). This excellent performance of bio-compatible RLs in speckle-free imaging might extend the RL applications in modern laser applications.

#### 4.1.2. Stretchable or Bendable RLs

**Figure 6:**
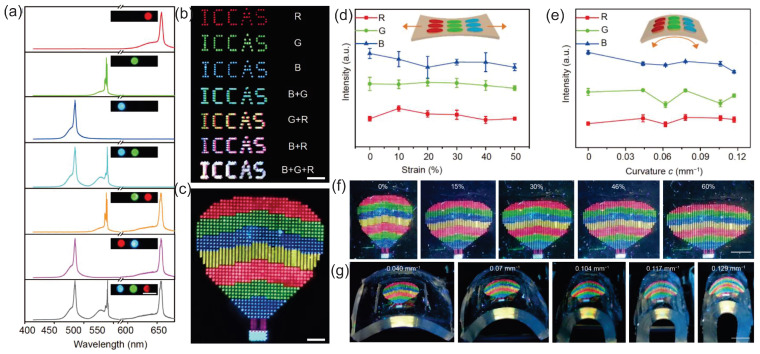
Full-color and pixelated flexible laser display. (**a**) Lasing spectra of different combinations of pixelated micro RLs. Three different Pe-NCs were used to provide RGB colors. (**b**) “ICCAS” patterns of different combinations of pixelated micro RLs. (**c**) A hot air balloon pattern from a multipixels array on a PDMS substrate. (**d**) Lasing intensities of the identical pixel at 658, 536 and 483 nm in terms of stretching strain values. (**e**) Lasing intensities of the identical pixel at 658, 536 and 483 nm in terms of bending curvature values. (**f**) Photographs of stretching multipixel display under different strains. (**g**) Photographs of bending multipixel display under different curvatures. Adapted from Ref. [43] with permission from Science China Press and Springer-Verlag GmbH Germany, part of Springer Nature.

The flexible laser displays and laser illumination devices can be achieved in RLs owing to the flexibility of random media [16,43,44,45,46]. Recently, a pixelated full-color flexible laser display was reported, in which, the pixelated RLs were precisely positioned into the well-arranged red–green–blue (RGB) arrays to form patterns such as “ICCAS” patterns shown in Figure 6b and a hot air balloon pattern shown in Figure 6c [43]. In detail, perovskite nanocrystals (Pe-NCs) and monodesoerse silica spheres serve as the gain medium and optical feedback, respectively. They were synthesized into composites Pe-NCs/SiO${}_{2}$ and dispersed in the solutions, which were further injected into the well-designed circular microtemplates with a depth of 10 µm. The author ruled out the feedback mechanisms of WGM and FP microcavities as the laser emission was undirectional and the mode spacing was inconsistent with the calculated FP cavity mode spacing.

The stability of the flexible laser display was also tested under mechanical deformations. The variations in the lasing intensity, wavelength and threshold are nearly negligible either under stretching, shown in Figure 6d,f, or under bending, shown in Figure 6e,g. In another bendable RL, the peak wavelength barely changed with different curvatures, too [45]. These results indicate that RLs are promising in the construction of portable and wearable laser displays or illumination devices.

#### 4.1.3. Wavelength-Tunable or Multicolor RLs

Not all RLs can keep stable under the mechanical deformations. This drawback for flexible laser displays in turn leads to the wavelength-tunable RLs, which are normally achieved with the fluorescent dyes. A wavelength blueshift of 14.54 nm was observed when the PET film, decorated with Ag nanoprisms and spin-coated with R6G film, was bent with a bending strain of 50% [46]. In another RL consisting of a silicone rubber slab that was press-coated with different thicknesses of R6G and Ag nanowires (NWs) film, a 17 nm redshift was obtained by excitation at locations with an increasing film thickness [48].

**Figure 7:**
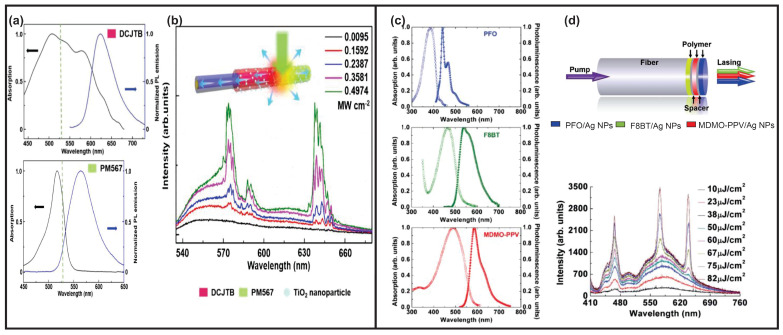
(**a**) The excitation and emission spectra of DCJTB film with a concentration of 1.5 mg/mL in PDMS and PM567 film with a dye concentration of 1.25 mg/mL in PDMS, both pumped with a Q-switched Nd:YAG laser with wavelength of 532 nm and pulse duration of 8 ns. (**b**) Schematic diagram of the red–yellow switchable fiber and the emission spectra of two-color RLs. Adapted from Ref. [47] with permission from Royal Society of Chemistry. (**c**) The excitation and emission spectra of PFO, F8BT and MDMO-PPV polymer. Adapted from Ref. [149] with permission from Royal Society of Chemistry. (**d**) Schematic diagram of an RGB RL on an optical fiber facet and the emission spectra of the RGB RLs. Adapted from Ref. [150] with permission from Royal Society of Chemistry under CC BY 3.0 license.

In other circumstances, such as multiple gain media, multicolor RLs can be achieved. However, the simultaneous excitation of multiple gain media with one single pump laser wavelength is an issue due to the limited selections of fluorescent dyes. To obtain multicolor RLs with a single excitation, on the one hand, gain media with the same excitation wavelength ranges but different emission wavelength ranges were chosen, such as 4-(dicyanomethylene)-2-tert-butyl-6-(1,1,7,7-tetramethyljulolidin-4-yl-vinyl)-4H-pyran (DCJTB) and Pyrromethene567 (PM567), shown in Figure 7a [47].

On the other hand, cascade pumping assists single-excitation multicolor RLs. Basically, in a cascade pumping, the emission of the former gain medium can excite the other gain media. In Figure 7c, three polymer materials, i.e., poly[(9,9-dioctylfluorenyl-2,7-diyl)-altco-(1,4-benzo-(2,1${}^{\prime }$,3)-thiadiazole)] (F8BT), poly[2-methoxy- 5-(3${}^{\prime }$,7${}^{\prime }$-dimethyloctyloxy)-1,4-phenylenevinylene] (MDMO-PPV) and poly[9,9-dioctylfluorenyl-2,7-diyl] (PFO), were used as gain media. The gain media were then mixed with Ag NPs solutions and dip-coated into the end face of the optical fiber in the sequence of the F8BT, MDMO-PPV and PFO layer (Figure 7d upper row) [149,150].

The sequence is important as the cascade energy transfer is inevitable. A pump laser with a wavelength of 400 nm was used to excite all of the gain media. Besides that, both F8BT and MDMO-PPV gain media can be excited by the emission from other gain media. Therefore, the F8BT and MDMO-PPV layers should be placed before the PFO layer during excitation to avoid too much of an absorption loss for the PFO gain medium. The MDMO-PPV layer was further placed closer to the pump light due to its higher damage threshold compared to the F8BT layer [150]. By varying the pump intensity, different chromaticity ranges of a white light mixed by RGB colors were achieved, as shown in Figure 7d lower row.

**Figure 8:**
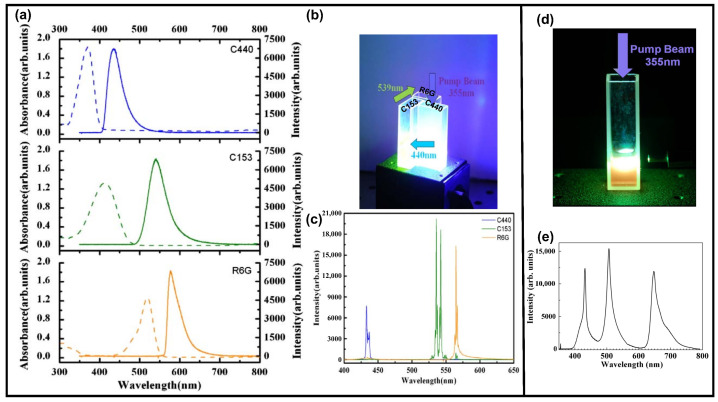
(**a**) The excitation and emission spectra of C440, C153 and R6G. (**b**) Pumping sequence for different gain media shown in (**a**). (**c**) The resultant RGB coherent feedback RL by cascade pumping shown in (**b**). Adapted from Ref. [151] with permission from Optical Society of America. (**d**) Single excitation of mixture solution of TiO${}_{2}$ NPs and gain medium of C6, C440 and oxazine. (**e**) The resultant RGB incoherent feedback RL generated from mixture solution shown in (**d**). Adapted from Ref. [152] with permission from American Institute of Physics.

A similar white RL with a coherent emission (Figure 8c) was also achieved by cascade pumping, but was built in liquid samples [151]. The mixture of Ag-Au bimetallic porous NWs and gain media of coumarin 440 (C440), coumarin 153 (C153) and R6G was prepared in three cuvettes, respectively. A Nd:YAG laser with a wavelength of 355 nm was used to pump the C440. The emission of C440 pumped the C153 and the emission of C153 pumped the R6G. The pumping sequence is shown in Figure 8b, and the excitation and emission spectra of each gain medium are shown in Figure 8a. In another random medium [152], the gain media of oxazine, coumarin 6 (C6) and C440 were mixed in a cuvette and the TiO${}_{2}$ NPs provided the optical feedback; see Figure 8d. Both C440 and C6 were pumped by a laser with a wavelength of 355 nm, and part of the emission of C6 pumped oxazine. The resultant white RL combined by RGB colors is shown in Figure 8e.

In a summary of Section 4.1, the RL-based light sources have advantages of bio-compatibility, flexibility and tunability, and facilitate in the speckle reduction in the laser-based imaging. Because of the widely available and easily manufactured scattering materials, the mass production of RL light source devices has a bright future in the market. In the display’s circumstance, the RL-based displays gain the advantages of laser properties, such as a high power-to-light conversion efficiency and narrow spectral range in comparison to conventional LED displays. The limitation might lie in the optical excitation. The electrically pumped gain media need to be further exploited.

### 4.2. RL as an Optical Sensor 

#### 4.2.1. RLs for Optomechanical and bio-Chemical Sensing

The sensing applications of RLs have been widely exploited. Basically, RL behaviors are sensitive to variations in random media, such as variations in gain media [13,53,93,118], scatterers [54,91,111] and the refractive index of the solvent [55,56,60]. Changes in the external environment, such as the humidity [153], temperature [8,154,155] and PH value [59], can also lead to variations in RL features. In addition, optomechanical strains have been detected through RL features both in biological hard tissues [26] and soft tissues [57].

**Figure 9:**
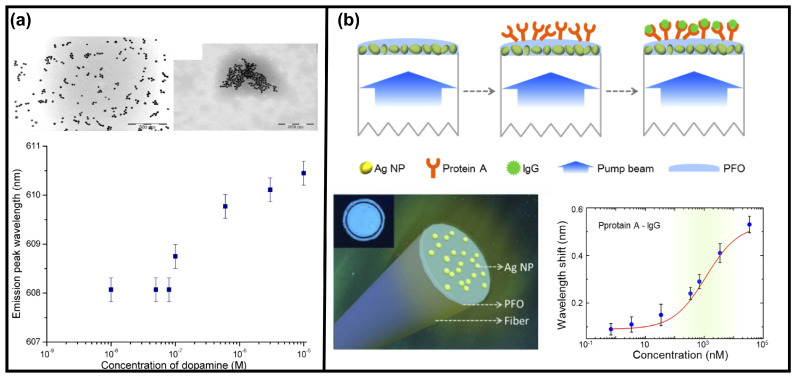
RLs used as biosensors. (**a**) Transmission electron microscopy of gold NPs with 0.15 mM copper (II) chloride. The aggregation of gold NPs is triggered by the combination of copper ions and dopamine as shown in the upper row right. The lower row graph shows the response of RL peak wavelength on the dopamine concentration. $10^{-4}$ M Rh640 was added as RL gain medium. Adapted from Ref. [58] with permission from the Optica Publishing. (**b**) Schematic diagram of capture immunoassay employed for IgG detection (upper row) and the design of RL on the fiber facet (lower row left). The lower row right graph shows the change in RL peak wavelength shift on the IgG concentration. Adapted from Ref. [60] with permission from the Optica Publishing under the terms of Open Access Publishing Agreement.

Another RL-based biosensor that can detect the dopamine concentration in a liquid sample dispersed with gold NPs and copper ions was reported [58]. The detection of dopamine concentration changes is important, as it is associated with brain diseases such as Parkinson and Huntington’s disease. In this report, variations in RL spectra, including the lasing threshold, peak wavelength, linewidth and peak emission intensity, were detected during the variations in the scattering strength caused by the aggregation of gold NPs. This aggregation was triggered by the combination of dopamine and copper ions, as shown in Figure 9a upper row right. In such a way, the aggregation degrees relate to the concentrations of dopamine. Therefore, the changes in dopamine concentration can be manifested as the changes in RL spectral features such as the peak wavelength, linewidth and peak intensity [58]. The change in the RL peak wavelength is shown in Figure 9a lower row, and a detection limit down to $1\times 10^{-7}$ M was achieved.

In another report, the RL biosensor was assembled for the fiber facet [60], as shown in Figure 9b lower row left. The fiber facet was dip-coated in the solution of Ag NPs and in the gain medium of dimethyl PFO in sequence. After being further assembled by the protein A (primary antibody), this biosensor was proposed to detect the concentrations of human immunoglobulin G (IgG) solution (another type of antibody that can bind with protein A through immunization interaction). The wavelength shift is sensitive to the IgG concentration, as shown in Figure 9b lower row right. Such an RL biosensor assembled in the fiber facet has advantages in portable and integrated detection devices.

#### 4.2.2. RLs for Tissue or Cell Differentiation

Considering that biological tissues are natural scattering materials and, moreover, different tissues exhibit different scattering strengths, RLs have been proposed to achieve tissue differentiation and even cell differentiation. In this respect, the corpus callosum region of brain tissues was revealed by the RL threshold [28]. In detail, a lower RL threshold was found in this region because it consists of a fiber structure and lipid rich compositions, which might lead to a stronger light scattering [28]. A rapid cytometry of apoptosis was also achieved using RL spectra through the PFT analysis. The scattering strength was varied as the cell morphology was deformed due to the drug treatment [61].

For other tissues or cells that exhibit similar RL behaviors, the differentiation remains a challenge. Thereby, in the report from Hohmann et al. [27], additional evaluation methods for RL spectra, such as a statistical analysis, were employed. This tissue differentiation is based on a classification of RL information using machine learning algorithms. The success of the tissue differentiation has potential applications in laser surgery to prevent wrong tissue cuttings.

The same issue occurred here [62], where the samples cannot be distinguished by the explicit RL properties, e.g., thelasing threshold. Therefore, a similar statistical analysis of RL spectra from the three types of cells, i.e., transfected cells that express the pathogenic and the non-pathogenic forms of the huntigtin (HTT) gene and the non-transfected cell, was conducted. The identification of cells that express a mutant HTT gene will provide benefits for the prevention of neurodegenerative disease such as Huntington’s disease, and might open novel applications in the early stages of clinical genetic testing.

#### 4.2.3. RLs for Cancer Diagnosis or Therapy

**Figure 10:**
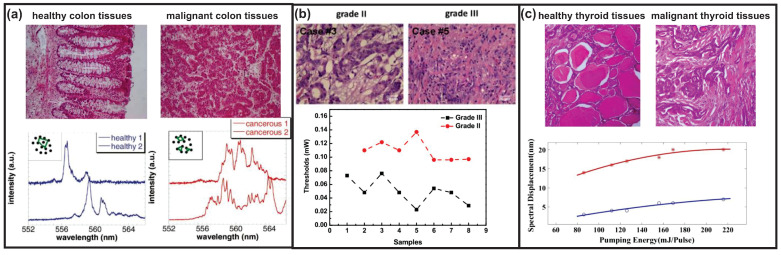
RLs used for cancer diagnosis. (**a**) Healthy and cancerous human colon tissues filtrated with R6G solution and the corresponding RL spectra. Adapted from Ref. [30] with permission from the AIP Publishing. (**b**) Different malignant grades of human breast tumor tissues stained with DCJTB solution and the thresholds of RLs generated from 15 randomly selected tumor tissues. Adapted from Ref. [32] with permission from Springer Nature under CC BY 4.0 license. (**c**) Healthy and cancerous human thyroid tissues filtrated with R6G solution and the corresponding RL peak wavelength shifts in terms of pumping energy. Open blue circles represent healthy tissues and red asterisks represent malignant tissues. Adapted from Ref. [31] with permission from Springer Nature.

Several RL spectral properties such as the lasing modes [30], threshold [32] and peak shift [31] were employed to distinguish between healthy and cancerous tissues. Polson et al. [30] reported RLs from R6G-infiltrated human colon tissues. RL spectra generated from healthy and cancerous tissues display obvious differences in the emission modes. There are more lasing modes in the RL spectra of cancerous tissues because of more disordered cells; see Figure 10a. Furthermore, using the numerical method of averaged PFT, the dominant cell sizes of healthy tissues were successfully calculated, while no well-defined cell sizes exist in the cancerous tissues.

Based on their contributions, Wang et al. [32] further exploited and found that different malignant grades of breast cancerous tissues can be specified through the RL threshold. As shown in Figure 10b, the RL threshold of malignant grade III tissues is always lower than that of malignant grade II tissues. This is probably because there are more disorders in malignant grade III tissues. In addition, a wavelength shift can also be applied for cancer diagnosis [31]. The cancerous thyroid tissues always induce a peak wavelength at the longer-wavelength side compared to the healthy thyroid tissues at the same pumping energy, as shown in Figure 10c. The author argued that light dwells longer due to more disorders in the cancerous tissues, resulting in a stronger reabsorption and re-emission effect, which causes a stronger peak wavelength redshift.

**Figure 11:**
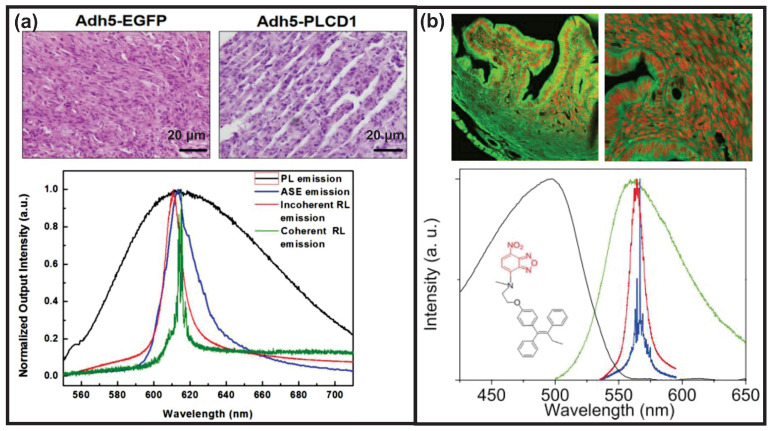
RLs used for cancer therapy. (**a**) Breast cancerous tissues without therapy (Adh5-EGFP) and with therapy (Adh5-PLCD1). The coherent feedback RL is obtained from Adh5-EGFP tissues, whereas the Adh5-PLCD1 tissues generate incoherent feedback RL. Adapted from Ref. [33] with permission from the AIP Publishing. (**b**) Confocal microphotographs of mouse uterine tissues labeled with FLTX1, which is a modified drug molecule displaying green fluorescence. The nuclei of cells are stained by a red fluorescent agent, i.e., the propidium iodide dye, in order to better observe the nuclear compartments. Both incoherent and coherent feedback RLs are obtained when moving the pump position. The chemical structure of FLTX1 is given as an inset in the spectra. Adapted from Ref. [29] with permission from the IOP Publishing.

Likewise, RLs are also feasible for cancer therapy as the cancerous tissues after therapy might show less disorder. This was evidenced with the breast cancerous tissues shown in Figure 11a [33]. A coherent feedback RL was obtained from breast cancerous tissues without therapy (Adh5-EGFP), whereas the cancerous tissues after therapy (Adh5-PLCD1) generated an incoherent feedback RL. It is worth noting that both cancerous tissues with or without therapy were infiltrated with the DCJTB dye solution, which was selected as the gain medium for RL emission.

In another strategy for cancer therapy, the drug molecule was modified to emit green fluorescence so that no additional dye was required for the generation of RLs [29]. Basically, the modified anticancer drug FLTX1 was formed by binding the fluorescent biomarker for lipid membranes to a well-known breast cancer drug tamoxifen (Tx). During the verification of this anticancer drug, both incoherent and coherent feedback RLs, as shown in Figure 11b lower row, were obtained by moving the pump positions. This revealed the spatially various disorders of the tissues. However, a further investigation of the therapy effect was not conducted.

In a summary of Section 4.2, all of the RL spectral properties—the lasing threshold, the peak wavelength shift and the appearance of the spectral modes—can be sensing parameters of an RL sensor. Under the precondition that the gain medium is kept unchanged, the principle behind all RL sensors is the same: the RL properties respond to the variations in the light diffusion, regardless of whether the variations are caused by the scattering particle aggregations, the changed concentrations, the intrinsic dielectric constant difference among different materials or environmental conditions such as the humidity, PH value, temperature and mechanical stress. One type of the RL sensors is manufactured to be isolated from the sensing targets, such as the prototype of fiber sensors with both the scattering material and gain medium assembled onto the fiber facet. This RL sensor platform is small, hand-held and disposable, considering that a massive production of fibers is not an issue nowadays. With biological materials such as tissues and cells as sensing targets, the materials themselves could be the scattering materials used to construct the RL sensors. The current factor lacking in RL sensing is the sensor performance characteristics; for example, the evaluation of stability or time-dependent degradation, linear or nonlinear response of the sensors and the corresponding calibration, sensitivity and accuracy measurements.

## 5. Summary and Outlook

This review showed how light can interact with disordered systems in RLs in rich and complex ways. Herein, only a systematic description of the most common RL emission properties and the influence factors was given according to the experimental observations, as summarized already in Table 1. Numerous modeling methods, such as the models of RL modes in Figure 2, could describe, predict and control the orders from the disorders. The understanding of a complex disordered system is still far away from being enough. This includes the understanding of the emerging RL physics; for instance, the stimulated Raman scattering as discussed in Section 3. Nevertheless, with the help of the RL, a partial description, prediction and control of the disordered systems were achieved, especially in the RL applications of the optical sensors and light sources.

The limitation of the RL applicability is recognized due to the relatively low Q-factor (or high threshold), as well as relatively low controllable lasing modes, lasing direction and polarization. To that end, design strategies of the pump light profile, such as adding disorders using a spatial light modulator, were proposed to reduce the threshold [85], to select the individual mode [80] and to control the predetermined lasing direction [156]. Another approach could be the utilization of an external platform such as a waveguide [157], FP microcavity [107,109] and WGM microcavity [158]. The combination of a scattering gain medium with a microfluid device brings RLs into a further stage of practical applications [34]. The idea behind the external platform is to alter the light scattering path, the features of which are reflected by the RL emission properties. In addition, an RL with a controlled threshold, coherence, direction and polarization can be achieved with liquid crystals. A certain order from disorder is realized by the easy alignment of liquid crystal, either by external electric or temperature control [16,159,160,161]. Another material, the Janus particle—which acts as a micro-heater once illuminated by an external laser—can change the assembly of scattering particles around it. An RL with a tunable laser threshold was achieved by the fully optical control from the external stimuli laser on the Janus particle [162]. Above all, one intriguing future research could be the design strategies of the pump profile and external platform for a given special RL scattering material to control the generation of random lasing from them.

RL is also a photonic platform for the fundamental statistical analysis of complex systems, as already acknowledged by the 2021 Nobel prize in physics [163]. Replica symmetry breaking, which reveals the phase changes and the interplay of system disorders and fluctuations, was experimentally demonstrated in RLs [99]. The replica symmetry breaking of the RL intensity fluctuation correlation was observed around the threshold pump intensity [99]. A further investigation could be the manipulation of the RL system disorders to change the occurrence of the replica symmetry breaking. In this context, RLs promise to be a benchmark for other complex systems; for instance, to predict and control how human activities affect climate change.

In future studies, more investigations of SRS in RLs would be another intriguing topic. The reconstruction of Raman spectroscopy with a higher sensitivity by means of RLs might be feasible. In other aspects, whether features of RL-SRS coupled lasing could reveal the scattering strength of disordered systems needs to be explored. Although Raman lines emerged in the RL spectra from highly fluorescent dyes have been considered so far, the observation of Raman lines from other materials might still be expected. In this regard, a dual-functional RL-SRS sensor—with RL signals detecting physical changes such as light diffusion and Raman signals detecting chemical changes such as molecular vibrations—is promising.

## Figures and Tables

**Table 1:** Different influence factors of the RL emission properties.

RL Emission Properties	Influence Factors	Experimental Parameters
Lasing threshold Tunability Coherence	Illumination	pump temporal profile (pulse duration) [78,79,80,81]
		pump spatial profile (size and shape) [14,54,80,82,83,84,85,86,87,88]
	Scatterers	concentration [7,15,53,54]
		size [89,90,91]
		shape [12,91,92]
	Dye	concentration [7,13,14,53,93]
	Solvent	refractive index difference [55,56]
	Collection	angles [21,47,60,94]

## Data Availability

No data are associated with this research work.

## Abbreviations

The following abbreviations are used in this manuscript:

RL	random laser
FP	Fabry–Pérot
Rh640	Rhodamine 640
WGM	whispering gallery mode
SF	superfluorescence
SL	superluminescence
ASE	amplified spontaneous emission
SR	superradiance
SRS	stimulated Raman scattering
RSB	replica symmetry breaking
NP	nanoparticle
RhB	Rhodamine B
R6G	Rhodamine 6G
PFT	power Fourier transform
RRL	random Raman laser
AF	Air Force
RGB	red–green–blue
Pe-NC	perovskite nanocrystal
NW	nanowire
DCJTB	4-(dicyanomethylene)-2-tert-butyl-6-(1,1,7,7-tetra-methyljulolidin-4-yl-vinyl)-4H-pyran
PM567	Pyrromethene567
F8BT	poly[(9,9-dioctylfluorenyl-2,7-diyl)-alt-co-(1,4-benzo-(2,1${}^{\prime }$,3)-thiadiazole)]
MDMO-PPV	poly[2-methoxy-5-(3${}^{\prime }$,7${}^{\prime }$-dimethyloctyloxy)-1,4-phenylenevinylene]
PFO	poly[9,9-dioctylfluorenyl-2,7-diyl]
C440	coumarin 440
C153	coumarin 153
C6	coumarin 6
IgG	immunoglobulin
HTT	huntigtin
Tx	tamoxifen

## Appendix A

**Table A1:** RL modes or Raman lines.

RL Medium	Pump Condition	Spectra	RL or Raman
ZnO nanospheres and R6G methanol colloid in flask	532 nm, 7 ns, 3 mm pump spot	Adapted from Ref. [140] with permission from the Elsevier.	R6G Raman lines
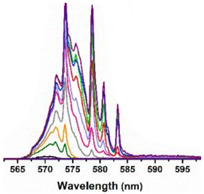
TiO${}_{2}$ and R6G ethanol colloid in cuvette	532 nm, 5 ns, 0.3 mm pump spot	Adapted from Ref. [141] with permission from the IOP Publishing.	R6G Raman lines
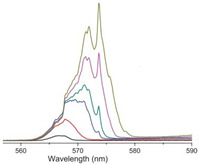
Ag nanowires and R6G methanol colloid in cuvette	532 nm, 10 ns, 3 mm pump spot	Adapted from Ref. [85] with permission from the American Chemical Society.	R6G Raman lines
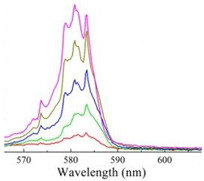
R6G and Ag nanowires ethanol colloid dispersed in a beaker	532 nm, 10 ns, 8 mm pump spot	Adapted from Ref. [164] with permission from the Optica Publishing.	R6G Raman lines
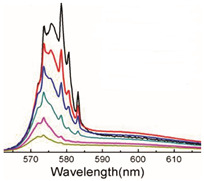
R6G doped PMMA acetone solution spin-coated onto hydrothermal oxidation treated Zn sheets	532 nm, 9 ns, 1 mm × 10 mm pump stripe	Adapted from Ref. [165] with permission from the Elsevier.	R6G Raman lines
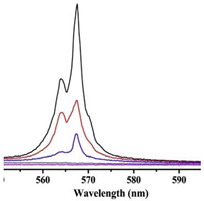
R6G and TiO${}_{2}$ urchins ethylene glycol colloid dispersed in a glass cuvette	532 nm, 120 ps, 2 mm pump spot	Adapted from Ref. [12] with permission from SPIE and the authors.	R6G Raman lines
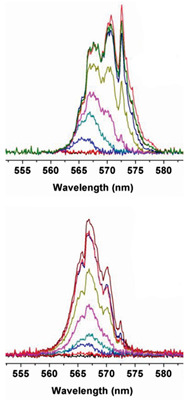
Au-Ag nanowires and R6G ethanol colloid in cuvette	532 nm, 8 ns	Adapted from Ref. [142] with permission from the American Chemical Society.	R6G Raman lines
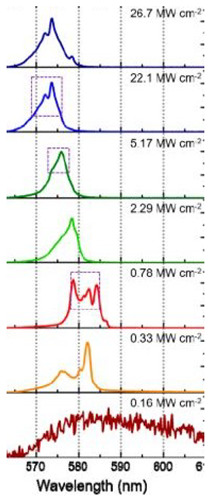
R6G methanol solution spin-coated onto a 1 µm thick ZnO nanowires integrated polyethylene terephthalate (PET) film	532 nm, 5 ns, 50 µm × 6 mm pump stripe	Adapted from Ref. [45] with permission from the Royal Society of Chemistry.	RL spikes and R6G Raman lines
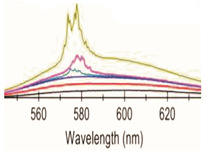
R6G and Au nanorods ethylene glycol colloid deposited between a reflecting plate and MgF${}_{2}$ plate	532 nm, 7 ns, 70 µm × 8 mm pump stripe	Adapted from Ref. [166] with permission from SPIE and the authors.	RL spikes and R6G Raman lines
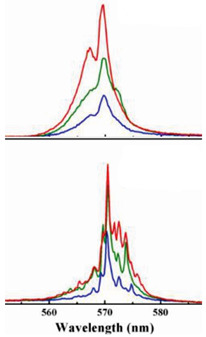
R6G and Ag nanowires ethanol polydimethylsiloxane (PDMS) mixture press-coated onto a silicone rubber slab	532 nm, 8 ns, 0.5 mm × 8 mm pump stripe	Adapted from Ref. [48] with permission from the Elsevier.	RL spikes and possible R6G Raman lines
